# Idiopathic basal ganglia calcification-associated *PDGFRB* mutations impair the receptor signalling

**DOI:** 10.1111/jcmm.12443

**Published:** 2014-10-08

**Authors:** Florence A Arts, Amélie I Velghe, Monique Stevens, Jean-Christophe Renauld, Ahmed Essaghir, Jean-Baptiste Demoulin

**Affiliations:** a De Duve Institute, Université catholique de LouvainBrussels, Belgium; b Ludwig Institute for Cancer Research, Brussels BranchBrussels, Belgium

**Keywords:** *PDGFRB*, Fahr disease, STAT3, receptor degradation, imatinib, brain calcification

## Abstract

Platelet-derived growth factors (PDGF) bind to two related receptor tyrosine kinases, which are encoded by the *PDGFRA* and *PDGFRB* genes. Recently, heterozygous *PDGFRB* mutations have been described in patients diagnosed with idiopathic basal ganglia calcification (IBGC or Fahr disease), a rare inherited neurological disorder. The goal of the present study was to determine whether these mutations had a positive or negative impact on the PDGFRB activity. We first showed that the E1071V mutant behaved like wild-type PDGFRB and may represent a polymorphism unrelated to IBGC. In contrast, the L658P mutant had no kinase activity and failed to activate any of the pathways normally stimulated by PDGF. The R987W mutant activated Akt and MAP kinases but did not induce the phosphorylation of signal transducer and activator of transcription 3 (STAT3) after PDGF stimulation. Phosphorylation of phospholipase Cγ was also decreased. Finally, we showed that the R987W mutant was more rapidly degraded upon PDGF binding compared to wild-type PDGFRB. In conclusion, PDGFRB mutations associated with IBGC impair the receptor signalling. PDGFRB loss of function in IBGC is consistent with recently described inactivating mutations in the PDGF-B ligand. These results raise concerns about the long-term safety of PDGF receptor inhibition by drugs such as imatinib.

## Introduction

Idiopathic basal ganglia calcification (IBGC), also called Fahr disease, is a rare neurological disease characterized by symmetrical bilateral calcifications, which are mostly located in the basal ganglia but can also be detected in other areas of the brain. The clinical features of this disease are heterogeneous and include a range of motor and psychiatric disorders such as dementia, parkinsonism, psychosis and seizures. IBGC is a familial disorder with an autosomal-dominant inheritance [1,2]. Until recently, not much was known about the physiopathology of the disease. Defects in the blood brain barrier integrity have been observed in one patient [3]. The breakthrough came only recently from genetic studies in patients and mice. Indeed, heterozygous mutations in three genes were associated with the disease: *SLC20A2*, *PDGFB* and *PDGFRB*.

Loss-of-function mutations were first identified in the sodium-dependent phosphate transporter gene *SLC20A2* [4–6]. Mice deficient in *SLC20A2* show brain calcification, indicating a causal relationship between *SLC20A2* mutations and the human disease [7]. Mutations were also found in *PDGFB*, which encodes platelet-derived growth factor B [8]. Although they were not characterized experimentally, these mutations were predicted to represent loss-of-function alleles. Homozygous *PDGFB* deletion in mice is lethal because of a severe lack of vascular smooth muscle cells and pericytes [9,10]. In mice carrying a viable *PDGFB* hypomorph allele, Keller and colleagues observed blood brain barrier abnormalities and brain calcification, which were reminiscent of human IBGC [8]. Interestingly, we have observed that platelet-derived growth factor (PDGF)-B increases the expression of *SLC20A2* in mesenchymal cell cultures, providing a possible link between these two genes in IBGC [11].

PDGF-B binds to two related receptor tyrosine kinases (RTK), PDGF receptor (PDGFR) α and β, which are encoded by the *PDGFRA* and *PDGFRB* genes [9,12]. PDGFR are divided into three parts: the extracellular domain which binds the ligand, the α-helix transmembrane domain and the intracellular kinase domain [13]. In the absence of ligand, the kinase domain is auto-inhibited by three cytoplasmic regions: the juxtamembrane domain, the activation loop of the kinase domain and the C-terminal tail [13–15]. Both α and β receptors stimulate cell proliferation and migration through an array of signalling pathways including mitogen-activated protein (MAP) kinases, phosphatidylinositol-3 kinase (PI3K), phospholipase Cγ (PLCγ) and signal transducers and activators of transcription (STAT) [12,16]. After ligand stimulation, the receptors are internalized and degraded in lysosomes [17].

Various types of genetic alterations activate these two receptors in cancers of mesenchymal and hematopoietic origin, such as *dermatofibrosarcoma protuberans*, gastrointestinal stromal tumours (GIST), inflammatory fibroid polyps, glioblastoma and myeloid neoplasms associated with hypereosinophilia [12,15,18]. A germline activating mutation in *PDGFRA* was associated with familial GIST [19]. Recently, *PDGFRB* mutations were also identified in autosomal-dominant infantile myofibromatosis, a disease characterized by myofibroblasts proliferation [20,21]. Contrasting with these cancer-associated mutations, Nicolas and colleagues found germline *PDGFRB* mutations in IBGC [2,22]. These are the first PDGF receptor mutations identified in a non-neoplastic disease. The L658P substitution was found in one family while R987W and E1071V were identified in sporadic cases [2,22]. The L658 residue is located in the intracellular kinase domain while R987 and E1071 belong to the C-terminal tail, which regulates the activity, signalling and endocytosis of the receptor [12,14]. The goal of the present study was to characterize the functional impact of these mutations on the receptor activity.

We show here that the PDGFRβ L658P mutant completely abolishes the receptor kinase activity and signalling while the R987W substitution increases the receptor degradation, leading to deficient STAT3 signalling. These observations complement the very recent report of Sanchez-Contreras *et al*. [23]. Finally, the E1071V mutant did not present defects consistent with an involvement in IBGC.

## Materials and methods

### Antibodies, reagents and constructs

Anti-PDGFRβ (958) and anti-phosphotyrosine (PY99) antibodies were purchased from Santa Cruz Biotechnology, Dallas, Texas, USA. The anti-PDGFRβ (AH 17.2) monoclonal antibody was produced as described [24]. Anti-phospho-STAT3 (Tyr705), anti-STAT3, anti-phospho-Akt (Ser473), anti-Akt, anti-phospho-PLCγ1 (Tyr783) and anti-PLCγ antibodies were purchased from Cell Signaling Technology, Danvers, MA, USA. Anti-calnexin antibody was purchased from Enzo® Life Sciences, Farmingdale, NY, USA. Secondary antibodies used in western blot experiments were purchased from Cell Signaling Technology. The secondary antibody coupled to phycoerythrin used in flow cytometry was purchased from Jackson ImmunoResearch, West Grove, PA, USA. Fluorescent secondary antibodies were purchased from LI-COR Biosciences, Lincoln, NE, USA. PDGF-BB was purchased from PeproTech, Rocky Hill, NJ, USA. Imatinib was purchased from LC Laboratories (Woburn, MA, USA). Cycloheximide was purchased from Sigma. ATP was purchased from Fermentas (Thermo Fisher Scientific, Waltham, MA, USA). EZ-Link Sulfo-NHS-Biotin and streptavidin agarose beads were purchased from Pierce (Thermo Scientific).

### Cell culture

The HT-1080 human fibrosarcoma cell line was a kind gift from Anabelle Decottignies (de Duve Institute, Brussels) and were cultured in Iscove's Modified Dulbecco's Medium (IMDM, Gibco, Life Technologies, Grand Island, NY, USA) supplemented with 10% foetal calf serum (FCS). MCF7 cells, a human breast adenocarcinoma cell line, and the human embryonic kidney HEK-293T cells were grown in DMEM (Gibco) supplemented with 10% FCS.

### Site-directed mutagenesis

Wild-type PDGFRβ was cloned in pcDNA3.1 (Invitrogen, Life Technologies). The point mutations L658P, R987W and E1071V were introduced by site-directed mutagenesis using the QuickChange™ XL-II kit as recommended by the manufacturer (Stratagene, La Jolla, CA, USA). All the constructs were verified by sequencing the whole insert.

### Luciferase assays

MCF7 cells were seeded in 6-well plates (300,000 cells/well). The day after, cells were cotransfected with wild-type PDGFRβ or its mutants (0.5 μg), a luciferase reporter controlled by serum-response elements (pSRE, 0.5 μg) and pEF1-β-galactosidase (0.5 μg) by using FuGENE® HD Transfection Reagent (Promega, Leiden, The Netherlands). Four hours after transfection, cells were washed with PBS and fresh medium was added. PDGF-BB (20 ng/ml) was added in the appropriate wells.

HT-1080 cells were seeded in 12-well plates (150,000–200,000 cells/well). The day after, cells were cotransfected with wild-type PDGFRβ or its mutants (0.5 μg), a plasmid in which the luciferase expression is controlled by a STAT1/3/5 sensitive promoter (pGRR5, 0.5 μg) and pEF1-β-galactosidase (0.5 μg) by using FuGENE® HD. Four hours after transfection, cells were washed with PBS and starved in minimum essential medium supplemented with 0.05% bovine serum albumin, 50 U/ml penicillin, 50 μg/ml streptomycin and 2 mM glutamine. Cells were stimulated with PDGF-BB (20 ng/ml) or left untreated.

Twenty-four hours after transfection, cells were lysed and the luciferase activity was assessed by using GloMax® as described [25]. The β-galactosidase activity was assessed by mixing 40 μl of lysate with 40 μl of substrate solution (165 mM Na_2_HPO_4_, 38 mM NaH_2_PO_4_, 2 mM MgCl_2_, 72 mM β-mercaptoethanol, 4.4 mM o-nitrophenyl-β-d-galactopyranoside) and by measuring the absorbance at 405 nm after 30 min. The results represent the ratio between luciferase and β-galactosidase activity. This ratio was set to 100% for stimulated wild-type PDGFRβ.

### Transfection, selection and sorting of cells expressing wild-type PDGFRβ or its mutants

HT-1080 cells were transfected by using FuGENE® HD. The cells were seeded in 6-well plates at 400,000 cells/well. The day after, the cells were transfected with PDGFRβ wild-type, L658P or R987W (0.5 μg/well). Four hours after transfection, cells were washed with PBS and fresh medium was added. Twenty-four hours after transfection, cells were transferred to 10 cm dishes and treated with 3 mg/ml G418. After amplification in culture, HT-1080 cells stably expressing wild-type PDGFRβ or mutants were sorted by flow cytometry. For this purpose, cells were first incubated with an anti-PDGFRβ (AH 17.2) antibody (5 μg/ml) for 40 min. at 4°C and then with a secondary antibody coupled to phycoerythrin for 40 min. at 4°C and in the dark. These cells were finally sorted by FACSAria III (BD Biosciences, Erembodegem, Belgium).

### Western blot

HT-1080 stably expressing wild-type PDGFRβ or the mutants were seeded in 6-well plates (400,000 cells/well). The day after, cells were starved overnight. Then, PDGF-BB was added in the appropriate wells for the indicated periods of time. After washing with PBS, cells were lysed with a lysis buffer (25 mM Tris, 0.15 M NaCl, 6 mM EDTA, 1.25 M glycerol, 1% Triton X-100, pH 7.4, 1.7 μg/ml aprotinin, 1 mM Pefabloc and 1 mM sodium orthovanadate). Total cell lysates were centrifuged at 16,000 × g for 20 min. at 4°C, separated by SDS-PAGE and transferred on polyvinylidene fluoride membranes. These membranes were then incubated with the appropriate primary antibody. When indicated, membranes were re-probed with another antibody after stripping (incubation in 0.4 M NaOH for 8 min.). To visualize the protein of interest, secondary antibodies coupled to horseradish peroxidase were used and membranes were incubated with Western Blotting Luminol Reagent Solution A and B (Santa Cruz Biotechnology).

### Immunoprecipitation and *in vitro* phosphorylation assay

HEK-293T cells were transiently transfected with wild-type PDGFRβ or mutants by using the calcium phosphate method as described [26]. Four hours after transfection, cells were washed with PBS and new medium was added. The day after, imatinib (1 μM) was added to the cells for 4 hrs. Cell lysates were produced as described above and incubated with an anti-PDGFRβ (AH 17.2) antibody overnight on wheel at 4°C and then with Protein A/G UltraLink Resin (Thermo Scientific) for 1 hr 30 on wheel at 4°C. Beads were washed twice with lysis buffer and once with the kinase buffer (50 mM HEPES pH 7.6, 50 mM MgCl_2_). Then, beads were incubated in 50 μl kinase buffer with or without 50 μM ATP for 10 min. at 30°C. The reaction was stopped by adding 4× Laemmli buffer. Western blotting experiments were performed as described above. Membranes incubated with an anti-phosphotyrosine antibody (PY99) were re-probed with an anti-PDGFRβ (958) antibody after stripping. To visualize the protein of interest, fluorescent secondary antibodies were used (Odyssey®; LI-COR Biosciences) and membranes were analysed with Odyssey® Infrared Imaging System (LI-COR Biosciences). Results were represented as the ratio between phospho-PDGFRβ and total PDGFRβ. This ratio was set to 100% for wild-type PDGFRβ incubated with ATP.

### Cell surface protein biotinylation

HT-1080 cells stably expressing wild-type PDGFRβ or R987W were seeded in 6-well plates. The day after, cells were starved in IMDM without FCS overnight. PDGF-BB (20 ng/ml) was added in the appropriate wells for 1, 2 or 4 hrs. Then, plates were placed on ice to prevent further endocytosis. After being washed twice in PBS pH 8, cells were incubated with 0.2 mg/ml sulfo-NHS-biotin (Pierce, Thermo Scientific) in PBS pH 8 for 1 hr on ice. Unbound biotin was inactivated by incubation in TBS (50 mM Tris, 150 mM NaCl, pH 8) for 5 min. Then, HT-1080 cells were washed twice with TBS and lysed as described above. Biotinylated surface proteins were immunoprecipitated with streptavidin agarose beads (Pierce, Thermo Scientific) and the presence of PDGFRβ was assessed by western blot.

## Results

### The L658P and R987W mutations alter PDGFRβ signalling while the E1071V mutation has no effect

We assessed the impact of the three mutations identified in patients diagnosed with IBGC (namely L658P, R987W and E1071V) on PDGFRβ signalling using a luciferase reporter assay as described [25]. MCF7 and HT-1080 cells were transiently transfected with the empty vector, wild-type PDGFRβ or one of the three mutants. Two signalling pathways known to be activated by wild-type PDGFRβ upon ligand stimulation were analysed by the use of two different reporter constructs: a promoter containing serum-response elements (pSRE) which are sensitive to MAP kinase activation, and a GRR5 promoter driven by STAT transcription factors [25].

**Fig. 1:**
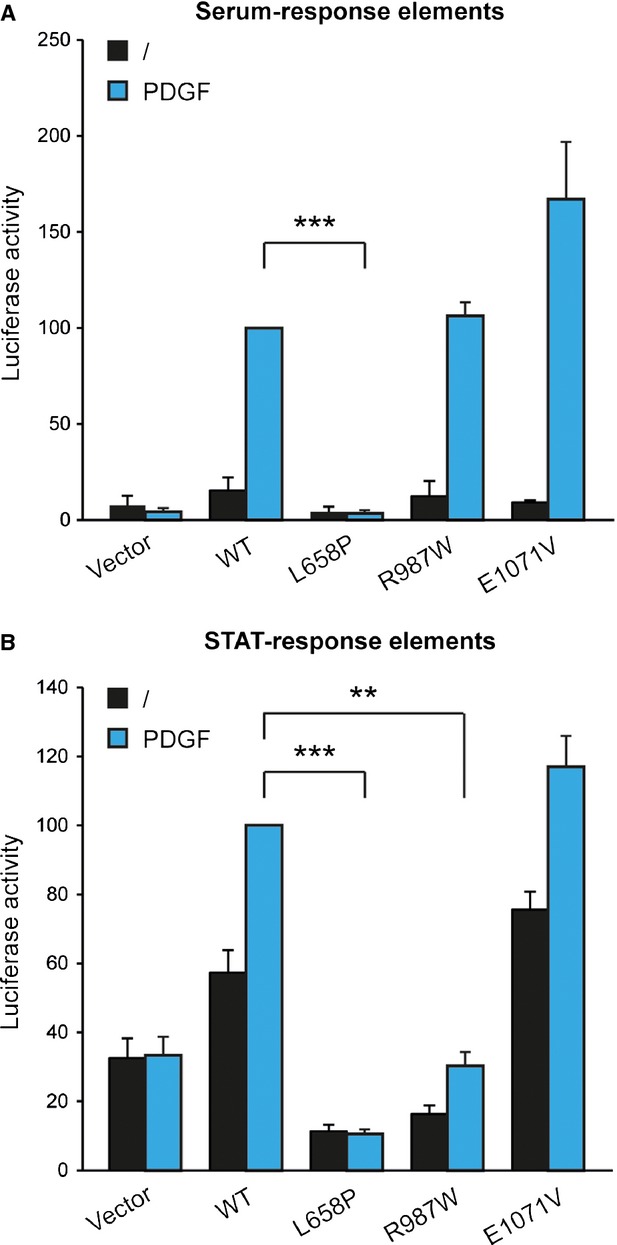
The mutations L658P and R987W, but not E1071V, impair PDGFRβ signalling. (**A**) MCF7 cells were transfected with plasmids containing the luciferase sequence downstream a SRE promoter, β-galactosidase and PDGFRβ wild-type (WT), L658P, R987W or E1071V. Four hours after transfection, cells were washed and treated or not with PDGF-BB (20 ng/ml) during 24 hrs. For each condition, luciferase activity was normalized with β-galactosidase activity. (**B**) HT-1080 cells were transfected with a STAT-driven (GRR5) luciferase reporter and processed as described above, except that cells were starved during the stimulation. In both cases, the average of three independent experiments is represented with SEM (Student's *t*-test; **P < 0.01; ***P < 0.001).

The two cell lines did not respond to PDGF, indicating that they did not express endogenous PDGF receptors (Fig. 1). As expected, PDGF activated both promoters in cells transfected with wild-type PDGFRβ. By contrast, the L658P mutant did not activate the two pathways. The R987W mutant was able to activate the MAP kinase reporter to the same extent as the wild-type receptor in response to PDGF, but a much decreased STAT response was observed. Unlike the two first mutations, E1071V did not affect PDGF-induced activation of the MAP kinase and STAT-driven reporters (Fig. 1). Thus, we did not analyse this mutant further.

Taken together, our results indicated that the L658P mutation abolishes PDGFRβ signalling, whereas R987W may specifically affect some signalling pathways.

### The L658P mutant is kinase-dead

**Fig. 2:**
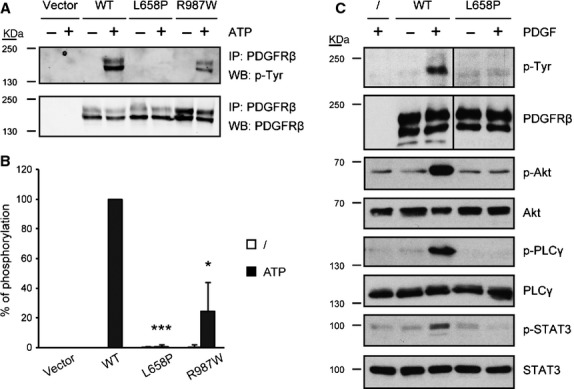
The L658P mutant is devoid of kinase activity. (**A**) 293T cells were transfected with PDGFRβ WT, L658P or R987W. After 20 hrs, cells were treated with imatinib (1 μM) for 4 hrs to prevent phosphorylation. Receptors were immunoprecipitated from cell lysates with an anti-PDGFRβ antibody and protein A/G beads. The beads were then incubated or not with ATP (50 μM) for 10 min. at 30°C. The phosphorylation level of the receptors was assessed by western blot using an anti-phosphotyrosine antibody. Then, the membrane was re-probed with an anti-PDGFRβ antibody. As a negative control, HEK-293T cells transfected with the empty vector (pcDNA3.1) were used. One representative experiment out of three is shown. (**B**) Quantification of the receptor phosphorylation level using an Odyssey instrument. The phosphorylation level was normalized with the total amount of receptor. The mean of three independent experiments is represented with SD (Student's *t*-test; *P < 0.05; ***P < 0.001). (**C**) Analysis of the phosphorylation and expression levels of the wild-type receptor and the L658P mutant as well as the phosphorylation of Akt, PLCγ and STAT3 upon ligand stimulation. HT-1080 cells stably expressing PDGFRβ WT or L658P were washed and starved overnight. Then, cells were treated or not with PDGF-BB (20 ng/ml) for 15 min. Cell lysates were analysed by western blot. After analysis of the phosphorylated protein, the membrane was re-probed with an antibody targeting the total amount of the corresponding protein. As a control, untransfected HT-1080 cells were used. Three independent experiments were performed with similar results. The vertical line indicates that lanes were cropped from the image of the same western blot membrane.

Given that the L658P mutation is located in the N-terminal lobe of the kinase domain and that this mutant showed no ability to activate signalling pathways in luciferase reporter assays, we tested if the substitution had a direct impact on the kinase activity of PDGFRβ. For this purpose, PDGFRβ was immunoprecipitated from transiently transfected HEK-293T cells and subjected to an *in vitro* kinase assay (Fig. 2A and B). In these settings, prior stimulation by PDGF is not required to activate the receptor [25].

Autophosphorylation of the wild-type and mutant receptors in presence of ATP was analysed by western blot (Fig. 2A). Although the L658P mutant was expressed to a similar level compared to the wild-type receptor, it was not phosphorylated on tyrosines in presence of ATP, demonstrating that it is devoid of kinase activity. By contrast, the R987W variant was phosphorylated but to a lesser extent compared to the wild-type receptor. Quantification of the blots presented in Figure 2A suggested that R987W decreased PDGFRβ phosphorylation by about 75% (Fig. 2B).

To further study these mutations, we stably transfected HT-1080 fibrosarcoma cells with wild-type or mutant receptors. The L658P mutant was expressed at a similar level compared to the wild-type receptor, as estimated by western blot (Fig. 2C), flow cytometry and quantitative PCR (Fig. S1). To confirm that L658P causes a loss of kinase activity, we studied signalling downstream this mutant by western blot (Fig. 2C). After PDGF stimulation, the L658P receptor was not phosphorylated on tyrosines (Fig. 2C), in agreement with the *in vitro* kinase assay results. Unlike the wild-type receptor, the L658P variant was not able to activate Akt, PLCγ or STAT3 upon ligand stimulation. We were not able to analyse Erk1/2 phosphorylation in response to PDGF because this pathway is constitutively activated in HT-1080 cells because of the presence of an active N-Ras mutant (data not shown).

Taken together, these results demonstrated that the L658P mutation completely suppresses the receptor kinase activity and signalling.

### The R987W mutation impairs activation of PLCγ and STAT3 by PDGFRβ

We further studied the R987W substitution in stably transfected HT-1080 cells. Although *PDGFRB* mRNA levels were comparable, the cell surface expression of the R987W mutant was slightly lower than wild-type or L658P, as shown by flow cytometry (Fig. S1). In addition, the quantification of different western blot experiments showed that R987W expression was reduced by 29% compared to the wild-type receptor (*n* = 13, Student's *t*-test, *P* < 0.001, data not shown). Phosphorylation of the mutant was similar to the wild-type receptor in transfected cells.

**Fig. 3:**
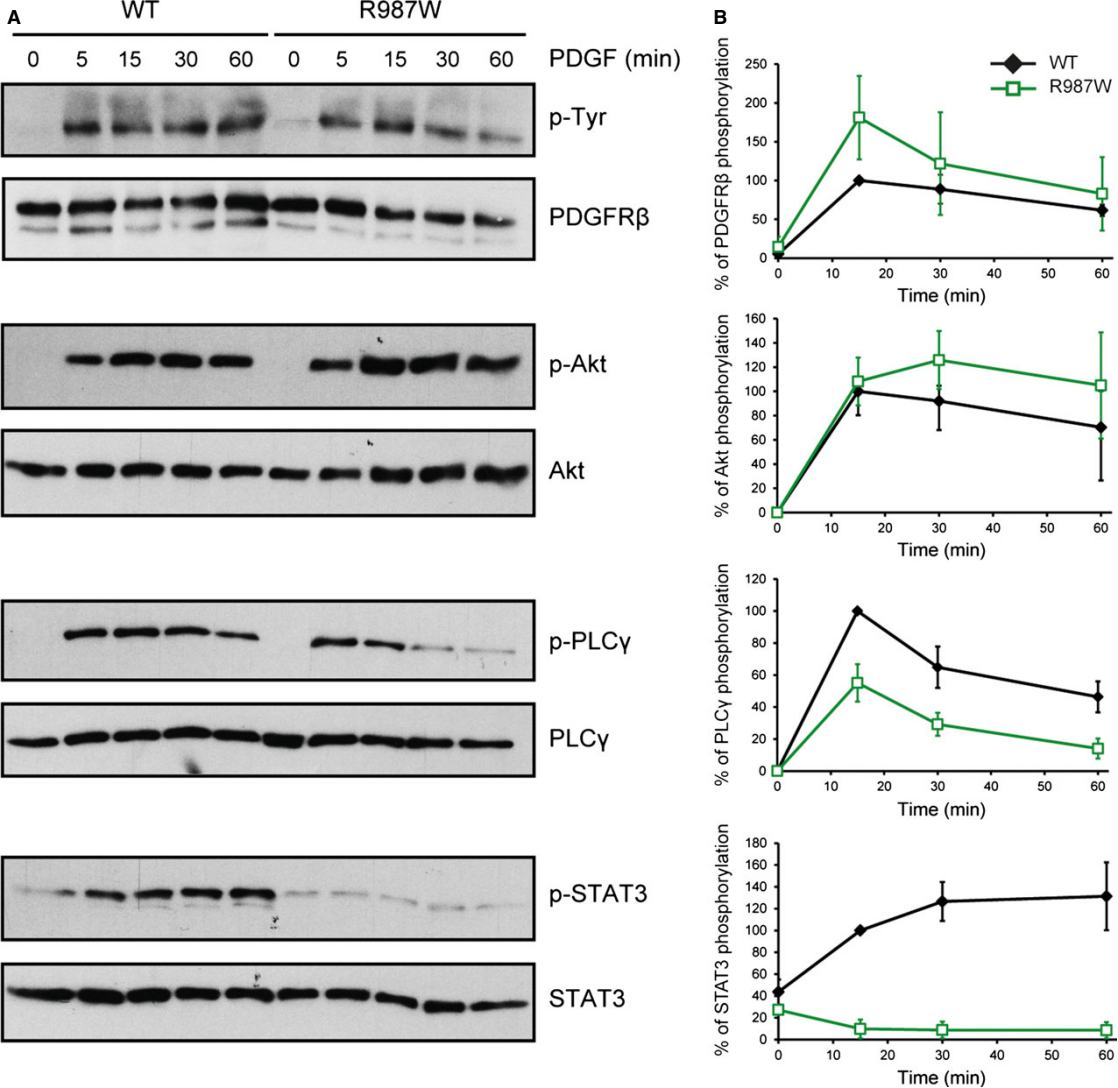
The R987W mutant does not activate STAT3. (**A**) Analysis of the phosphorylation and expression levels of the wild-type receptor and the R987W mutant as well as the phosphorylation of Akt, PLCγ and STAT3 upon ligand stimulation. HT-1080 cells stably expressing PDGFRβ WT or R987W were washed and starved overnight. Then, cells were treated or not with PDGF-BB (20 ng/ml) for 5, 15, 30 and 60 min. Cell lysates were analysed by western blot. After analysis of the phosphorylated protein, the membrane was re-probed with an antibody targeting the total amount of the corresponding protein. One representative experiment out of three is shown. (**B**) The percentage of PDGFRβ, Akt, PLCγ and STAT3 phosphorylation was quantified using the ImageJ software. The level of phosphorylation was normalized with the expression of the corresponding protein. The phosphorylation level of each protein was set to 100% for PDGFRβ WT-expressing cells stimulated with PDGF during 15 min. The average of three independent experiments is shown with SD (anova, *P* < 0.05 for p-PDGFRβ, not significant for p-Akt, *P* < 0.001 for p-PLCγ, *P* < 0.001 for p-STAT3).

Our results showed no difference in Akt activation by the two receptors, whereas PLCγ phosphorylation was decreased by about 50% (Fig. 3B). Strikingly, the R987W mutant failed to activate STAT3 in response to PDGF stimulation (Fig. 3A and B), consistent with our luciferase results.

Altogether, our results demonstrated that mutation R987W selectively impairs PDGFRβ signalling, in particular the activation of STAT3 and, to a lesser extent, PLCγ but not Akt.

### The R987W mutation increases PDGFRβ degradation upon PDGF stimulation

We noticed that the R987W mutant was degraded slightly faster in response to PDGF after one hour of stimulation (Fig. 3A). In addition, the R987W mutation is located about 20 residues downstream a leucine-rich region that has been involved in ligand-mediated endocytosis [27].

**Fig. 4:**
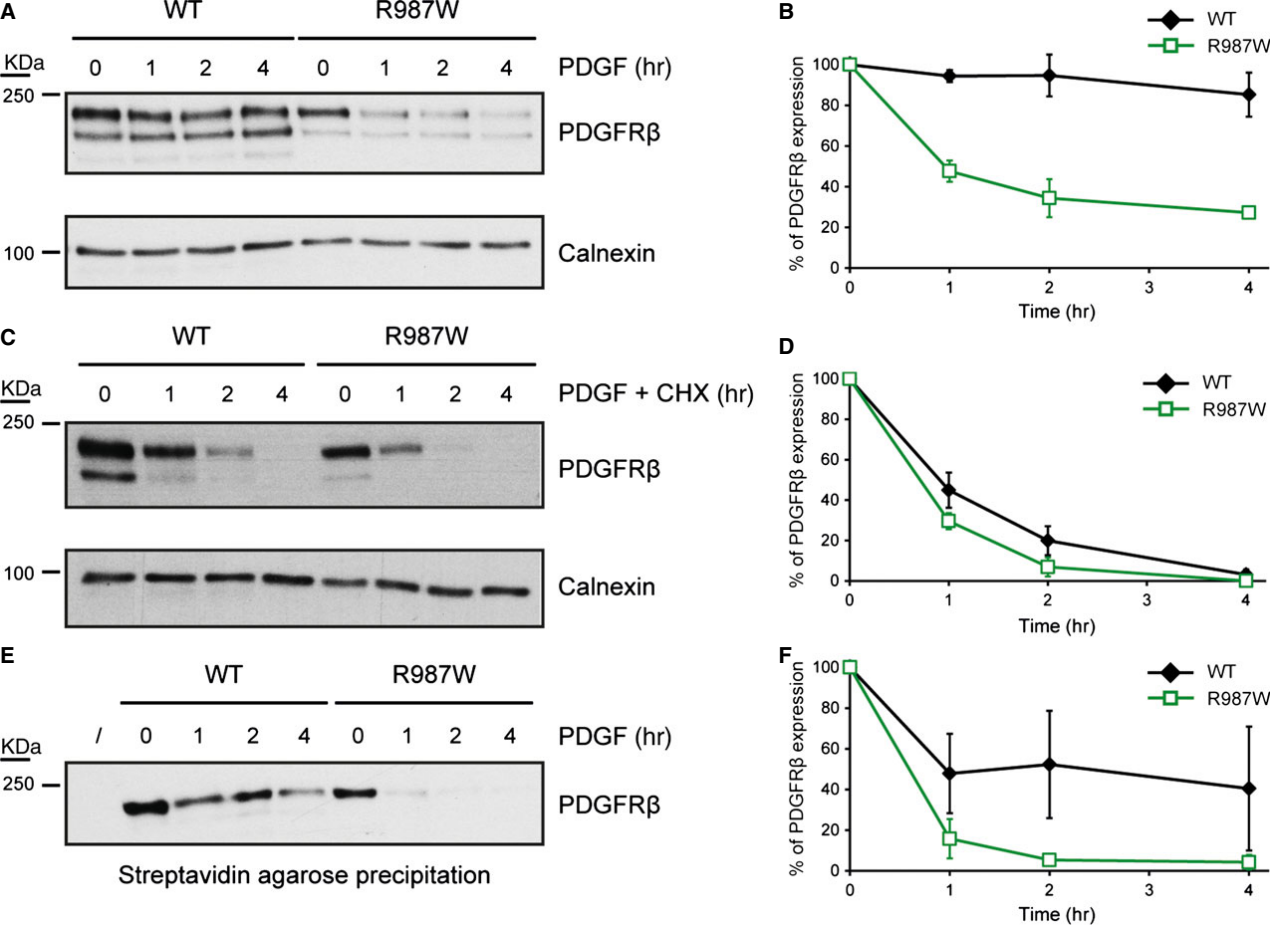
The R987W mutant is more rapidly internalized and degraded than the wild-type receptor. (**A**) HT-1080 cells stably expressing PDGFRβ or the R987W mutant were washed and starved overnight. Then, cells were treated with PDGF-BB (20 ng/ml) for 1, 2 or 4 hrs or left untreated. Cell lysates were analysed by western blot with an anti-PDGFRβ antibody. As a loading control, the membrane was re-probed with an anti-calnexin antibody. Three independent experiments were performed and a representative one is shown here. (**B**) The amount of PDGFRβ was quantified by using the ImageJ software and was normalized with calnexin expression. The average of three independent experiments is represented with SD (anova, *P* < 0.001). (**C**) The same experiment as in A was performed, except that cells were treated with cycloheximide (CHX, 50 μg/ml) in addition to PDGF (20 ng/ml). (**D**) The amount of PDGFRβ was quantified by using the ImageJ software and was normalized with calnexin expression. The average of three independent experiments is represented with SD (anova, *P* < 0.001). (**E**) HT-1080 cells stably expressing PDGFRβ or the R987W mutant were washed, starved overnight and treated or not with PDGF-BB (20 ng/ml) during 1, 2 or 4 hrs. Cells were incubated with sulfo-NHS-biotin and biotinylated cell surface proteins were precipitated with streptavidin agarose. The presence of PDGFRβ was assessed by western blot. One representative experiment out of three is shown. (**F**) Quantification of the amount of cell surface PDGFRβ by using the ImageJ software. The average of three independent experiments is shown with SD (anova, *P* < 0.001). In each quantification (**B**, **D** and **F**), the PDGFRβ amount was set to 100% for unstimulated PDGFRβ WT- and R987W-expressing cells.

To study the impact of R987W on the receptor degradation kinetics, HT-1080 cells stably expressing wild-type PDGFRβ or R987W were stimulated with PDGF for up to 4 hrs and the receptor expression level was assessed by western blot (Fig. 4A and B). We observed that the R987W mutant was quickly degraded after PDGF stimulation compared to wild-type PDGFRβ. When cycloheximide was used to block the re-synthesis of the receptor, this difference was still visible even though it was attenuated (Fig. 4C and D).

To more precisely assess the level of cell surface receptor, we performed cell surface protein biotinylation after treatment with PDGF. Biotinylated proteins were precipitated with streptavidin and analysed by western blot. Our results suggested that the R987W mutant was more rapidly internalized compared to the wild-type receptor (Fig. 4E and F).

In conclusion, these results showed that R987W is more rapidly degraded upon ligand binding in comparison with the wild-type receptor.

## Discussion

In this study, we demonstrated that the *PDGFRB* mutations that have been identified in IBGC lead to a partial or complete loss of function.

Our results showed that the L658P substitution completely abolished PDGFRβ activity. This was observed in three different cell lines (MCF7, HT-1080 and HEK-293T) in luciferase reporter assays, *in vitro* kinase assays and signalling experiments. This mutation has been identified in all IBGC-diagnosed members of one French family and was predicted to be damaging [2]. The L658 residue is highly conserved in PDGFRβ from different species and in PDGF receptor family members. It is located in the N-terminal lobe of the receptor kinase domain, in which the classical kinase-dead mutation K634A was also described [28]. The association of *PDGFRB* loss of function with IBGC is consistent with a reported role of PDGFRβ in cognitive and socio-emotional disorders based on the analysis of conditional *PDGFRB* knock-out mice [29]. Our results are also consistent with previous studies indicating that loss-of-function mutations in *PDGFB*, which encodes the PDGFRβ ligand, cause brain calcifications in mice and IBGC in humans [8,30]. Altogether, our results indicate that the L658P mutant encodes a kinase-dead receptor and confirm that it is responsible for IBGC, as suggested by Sanchez-Contreras *et al*. [23].

In contrast to L658P, we showed that the E1071V mutation, which was found in only one sporadic patient, has no negative impact on PDGF-induced activation of MAP kinases and STAT in luciferase assays. This residue has been poorly conserved during evolution but was predicted to be possibly damaging by Polyphen2 and deleterious by SIFT. It is absent from SNP databases. Nevertheless, it was predicted to be a polymorphism by Mutation Taster, which is compatible with our results [22]. The E1071V residue is located in a proline- and glutamic acid-rich region of the C-terminal tail that we have previously characterized as a negative regulator of the receptor activity. It is involved in the auto-inhibition of the receptor in absence of ligand [14]. If this model is correct, E1071V could increase the receptor activity. Indeed, in our luciferase reporter assays, the MAP kinase reporter was more strongly activated by E1071V than by wild-type PDGFRβ, although this did not reach statistical significance. Taken together, these data do not support the hypothesis that the E1071V mutation causes IBGC.

Unlike the L658P substitution, the R987W mutation, which is located outside the kinase domain, did not abolish the kinase activity. Nevertheless, this activity was decreased in an *in vitro* assay. However, this decrease was not confirmed in intact cells, as the tyrosine-phosphorylation of the mutant receptor was not affected in the HT-1080 cell line and signalling *via* Akt and MAP kinases was unchanged.

After PDGF stimulation, R987W was more rapidly internalized and degraded than the wild-type receptor. In addition, in the absence of exogenously added PDGF, the R987W receptor was expressed at a slightly lower level in HT-1080 cells, as shown by flow cytometry and western blot experiments. This was not because of a difference in transfection efficiency as mRNA levels were similar. This was not observed in transiently transfected HEK-293T or HeLa cells (Fig. 2A and data not shown). These results may be explained by previous reports showing that HT-1080 cells constitutively secrete PDGF [31]. Accordingly, we observed a slight constitutive phosphorylation of the receptors in western blot experiments (data not shown). These results apparently contradict a recent report by Sanchez-Contreras *et al*., who suggested that the R987W mutation severely decreased the receptor expression in HeLa cells in the absence of exogenous PDGF [23]. This might be related to the use of cell lines of different origins. Finally, the mechanism whereby this substitution affects the receptor degradation remains to be established. Noteworthy, the R987 residue, which is conserved in different species of mammals and fish as well as in FLT3, is located downstream a region that was identified as important for PDGFRβ ligand-mediated endocytosis [27]. Indeed, the deletion of this region abolished the receptor internalization after PDGF stimulation. Thus, a mutation near this region could affect PDGFRβ endocytosis [27]. In addition, the C-terminal tail is involved in the recruitment of c-CBL (Casitas B-lineage lymphoma) through a mechanism that remains controversial [32,33]. c-CBL is an ubiquitin ligase involved in PDGFR endocytosis and degradation through lysosomes. The R987W substitution could favour the activation of c-CBL, leading to an increase in receptor degradation [32].

Strikingly, the R987W mutation selectively impaired STAT3 activation by PDGF. The mechanism of STAT3 activation by PDGF remains elusive [12]. In particular, no direct docking site has been described in the receptor. Interestingly, one study suggested that STAT3 activation by PDGF requires the receptor internalization, allowing efficient STAT3 phosphorylation from endosomes [34]. This was also reported for other RTK such as Met [35]. Therefore, we hypothesize that R987W may prevent STAT3 activation by perturbing the receptor internalization and degradation pathway. Similarly, PLCγ phosphorylation, which was reduced by the R987W mutation, can also occur in endosomes [36]. Furthermore, PLCγ binds to residues Y1009 and Y1021, in the C-terminal tail [16]. The R987W substitution could cause a change in the C-terminal tail conformation that could decrease its accessibility or its phosphorylation on these tyrosines. Y1021 is also one of the proposed binding sites for FER, a tyrosine kinase that was recently involved in STAT3 activation [37]. In conclusion, the R987W mutation in the C-terminal tail could selectively affect the docking of PLCγ and FER to the PDGFRβ receptor, explaining the impact of this variant on some but not all PDGFR signalling pathways.

An important function of STAT3 downstream the PDGF receptor β was suggested in vascular smooth muscle cells, which are likely to play a key role in IBGC. Indeed, STAT3 activation is necessary for the expression of the cytosolic phospholipase A2 and, subsequently, for cell growth and motility [38,39]. In another report on the same cell type, it was shown that STAT3 inhibition by glutathione S-transferase is followed by a decrease in PDGF-induced proliferation [40]. These reports suggest a mechanism whereby a specific STAT3 impairment could link *PDGFRB* loss-of-function mutations to IBGC.

Idiopathic basal ganglia calcification is the first disease caused by loss-of-function PDGFR mutations, by contrast to the numerous activating mutations described in cancer-related disorders. Somatic PDGFRA loss-of-function mutations were recently characterized in cancers, but they likely represent passenger mutations that are not clinically relevant [25]. Interestingly, the three variants (L658P, R987W and E1071V) are located outside the two main hot spots of activating point mutations involved in cancer: the juxtamembrane domain and the activation loop [15]. However, L658 residue is located in a region where novel PDGFRβ point mutations were identified in infantile myofibromatosis [20,21]. These mutations have not been functionally studied yet but are likely to increase PDGF receptor signalling.

Currently, patients with GIST or myeloid neoplasms involving PDGFR gene alterations are successfully treated with imatinib [15,18]. Imatinib and other tyrosine kinase inhibitors are also administered for chronic myeloid leukaemia and other neoplasms. Relapse frequently occurs upon treatment discontinuation and patient should keep taking imatinib for very long periods of time [41,42]. Since the introduction of the drug, some patients have been treated for more than 10 years. The observation that inactivation of PDGFRβ can lead to brain calcification and IBGC raises concerns about the long-term safety of tyrosine kinase inhibitors that target this receptor. To our knowledge, symptoms like those observed in IBGC have not been documented yet but this should be carefully monitored in the future.

In conclusion, we showed that *PDGFRB* mutations associated with IBGC affect the receptor activity, degradation and signalling. These results support the current hypothesis that a defect in the PDGF-B/PDGFRB pathway can cause IBGC. Furthermore, these findings have consequences for the follow-up of long-term treatment with tyrosine kinase inhibitors and could pave the way for the development of a treatment for IBGC.

## Supporting information

**Figure S1** Stable expression of PDGFRβ in HT-1080 cells.
