# Supplementary material for: Idiopathic basal ganglia calcification-associated PDGFRB mutations impair the receptor signalling
Source: J Cell Mol Med. 2014 Oct 8;19(1):239–48. doi: 10.1111/jcmm.12443 (PMC4288366; doi:10.1111/jcmm.12443)
Supplement: Supplementary file 1 — Figure S1 Stable expression of PDGFRβ in HT-1080 cells. [file jcmm0019-0239-sd1.pdf]

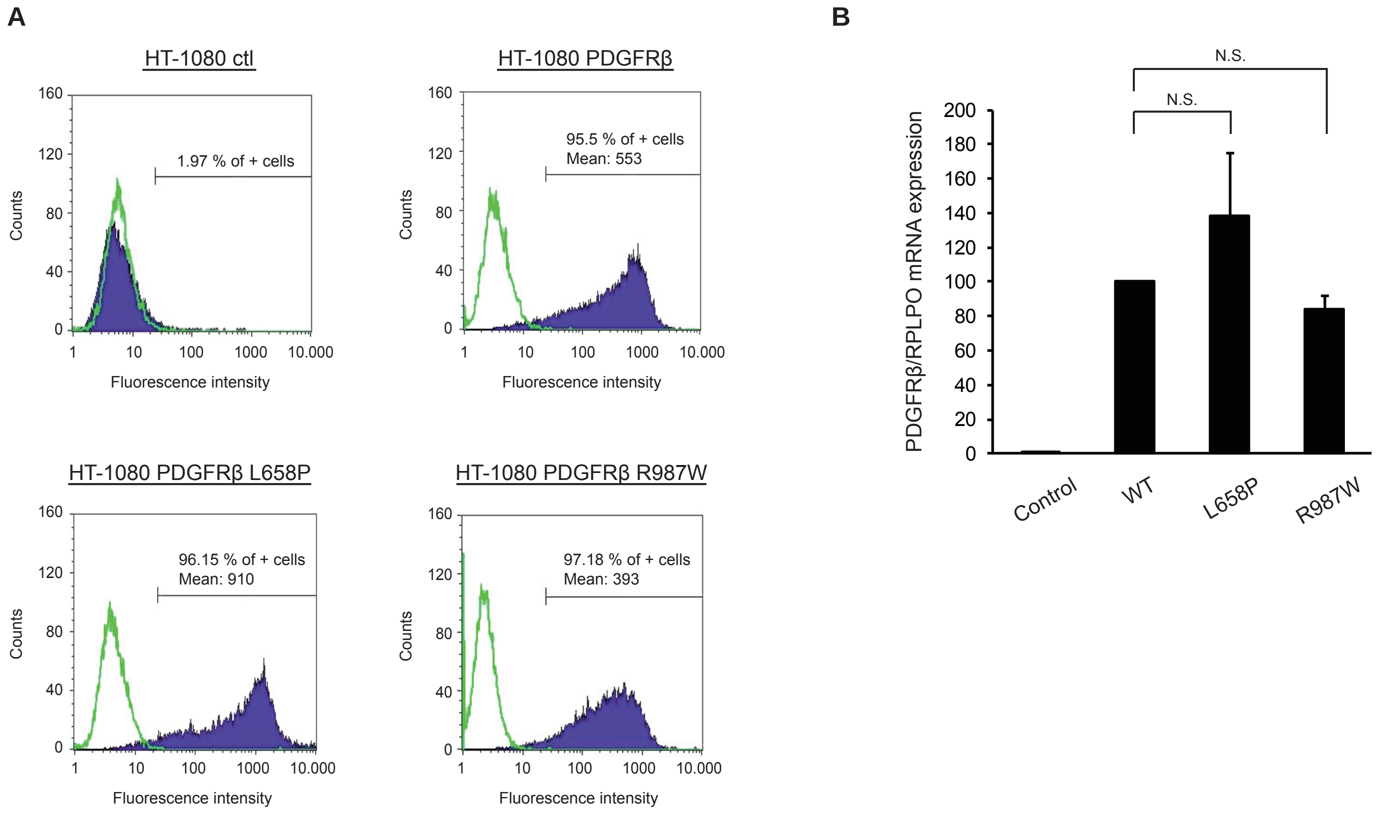

Suppl. Figure 1: Stable expression of PDGFRβ in HT-1080 cells.

A. Expression of the wild-type and mutant receptors at the plasma membrane. HT-1080 cells stably expressing the wild-type receptor, the L658P mutant or the R987W mutant were subjected to flow cytometry. To assess the percentage of HT-1080 cells that effectively expressed wild-type or mutant PDGFRβ, 500 000 cells were stained by using anti-PDGFRβ (AH 17.2) primary antibody (5 µg/ml) for 40 minutes at 4°C and a secondary antibody coupled to phycoerythrin (purple histograms) for the same period of time. As a negative control, cells were only incubated with the secondary antibody (green line). The cells were then analyzed by flow cytometry using a FACSCalibur instrument. One experiment out of two is represented.

B. mRNA level of the wild-type and mutant receptors. RNA was extracted from subconfluent HT-1080 cells stably expressing wild-type or mutant PDGFRβ with RNeasy Mini Kit (Qiagen). RNA (1 µg) was reverse transcribed by using M-MLV (Moloney Murine Leukemia Virus) reverse transcriptase (Invitrogen). To assess the level of PDGFRB mRNA, quantitative PCR was performed with 2 µl of cDNA and 23 µl of Absolute QPCR SYBR Green Fluorescein Mix 1x containing 70 nM of each of the following primers: GACCCCAAACCCGAGGTT and ATGGTTGAGGAGGTGTTGACTT. For each sample, qPCR was performed in triplicate. The results were represented as a ratio between PDGFRB mRNA level and RPLPO mRNA level. This ratio was set to 100% for wild-type PDGFRB. As a control, untransfected HT-1080 cells were used. The mean of three independent experiments is shown with S.E.M. (Student t-test; N.S., not significant).
